# Isotropically resolved label-free tomographic imaging based on tomographic moulds for optical trapping

**DOI:** 10.1038/s41377-021-00535-4

**Published:** 2021-05-17

**Authors:** Moosung Lee, Kyoohyun Kim, Jeonghun Oh, YongKeun Park

**Affiliations:** 1 Department of Physics, Korea Advanced Institute of Science and Technology (KAIST), Daejeon, 34141 South Korea; 2 KAIST Institute for Health Science and Technology, KAIST, Daejeon, 34141 South Korea; 3 Tomocube Inc., Daejeon, 34109 South Korea; 4 Present Address: Max Planck Institute for the Science of Light & Max-Planck-Zentrum für Physik und Medizin, 91058 Erlangen, Germany

**Keywords:** Microscopy, Optical physics

## Abstract

A major challenge in three-dimensional (3D) microscopy is to obtain accurate spatial information while simultaneously keeping the microscopic samples in their native states. In conventional 3D microscopy, axial resolution is inferior to spatial resolution due to the inaccessibility to side scattering signals. In this study, we demonstrate the isotropic microtomography of free-floating samples by optically rotating a sample. Contrary to previous approaches using optical tweezers with multiple foci which are only applicable to simple shapes, we exploited 3D structured light traps that can stably rotate freestanding complex-shaped microscopic specimens, and side scattering information is measured at various sample orientations to achieve isotropic resolution. The proposed method yields an isotropic resolution of 230 nm and captures structural details of colloidal multimers and live red blood cells, which are inaccessible using conventional tomographic microscopy. We envision that the proposed approach can be deployed for solving diverse imaging problems that are beyond the examples shown here.

## Abstract (web summary)

A general method for the in-situ isotropic microtomography of freestanding specimens, exploiting complex wavefront shaping and optical tweezers.

## Graphical abstract

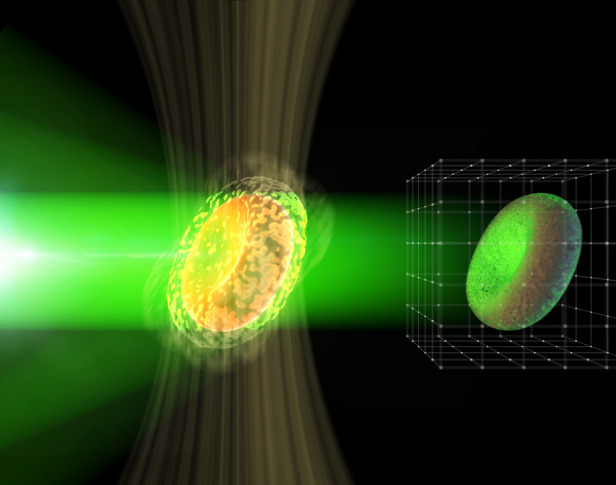

## Introduction

Improving the three-dimensional (3D) spatial resolution is a fundamental challenge in modern microscopy. Although most imaging modalities record 3D information with sub-micrometre resolution via axial^1^ or illumination scanning^2^, their axial resolution is inferior to the lateral resolution due to the finite numerical aperture (NA) of a condenser and an objective lens. This long-standing challenge is known as the missing cone problem because the Fourier spectrum of a reconstructed tomogram contains no information in a conical region along the optical axis^3^. The missing cone problem particularly impedes the accurate evaluation of axially thin 3D samples, such as rod-like microcrystals^4^, red blood cells (RBCs)^5,6^, and bacteria^7^.

To improve the axial resolution of 3D microscopy, various methods of sample rotation have been demonstrated. A straightforward approach is the rotation of a sample loaded in a microcapillary^8,9^ or a rotating tip^10,11^. However, the instrumentation for loading the specimen requires customised sample stages which reduce throughput and sample fixation which limits live-cell imaging. Another approach relies on the flow-assisted rolling of samples in microfluidic channels^12,13^. However, the precise tracking of the rapidly moving sample is computationally burdensome and incompatible with the in situ analysis of microscopic specimens.

In contrast, holographic optical tweezers^14^ (HOTs) allow in situ, all-optical control of the sample rotation, which may provide isotropic resolution in tomographic imaging without the use of customised sample stages^15,16^. Previous studies have exploited the feasibility of employing multiple focal beams to rotate cylindrically symmetric specimens^17–19^. However, these light traps often exert uneven force fields and result in imprecise rotations and deformations of the samples, thereby limiting their applications. Optimally structured light is as such indispensable for stable rotations and successful isotropic microtomography of general complex-shaped specimens, such as blood cells^20,21^.

Recently, our group has established that tomographic moulds for optical trapping (TOMOTRAP) could manipulate arbitrarily shaped freestanding objects and control their orientations^22^ using holographic traps designed using refractive-index (RI) tomograms. In this paper, we exploited TOMOTRAP for in situ isotropic tomographic imaging of freestanding microparticles. As a proof of concept, we imaged colloidal multimers for validation, followed by live mouse RBCs which are challenging to rotate without deformation. The resultant tomograms showed no missing cone artefacts as evidenced by the well-resolved spherical structures of colloidal particles and the biconcave dimples of RBCs, with more than doubled axial resolution.

## Results

### Principle of TOMOTRAP

TOMOTRAP optically manipulates and controls an arbitrarily shaped 3D sample by creating optimised 3D trapping light. The trapping light is optimised to resemble the 3D RI distribution of an optically trapped sample, *n*(**r**). In this condition, the electromagnetic field energy is maximised, and the optical trapping is most stable according to the electromagnetic variational principle^22^. To understand the maximisation condition, we assume the scalar diffraction theory in the weak scattering regime. If the scattered field is negligible compared to the incident field, *E*_in_(**r**). the time-averaged electromagnetic field energy is approximately

1 $$U_{{\mathrm {field}}} \approx \frac{{\varepsilon _0}}{2}{\int} {n^2({\mathbf{r}})|E_{{\mathrm {in}}}({\mathbf{r}})|} ^2{\mathrm {d}}{\mathbf{r}}$$

where *ε*_0_ is the vacuum permittivity^23,24^. The optical trapping is targeted only at a sample. Thus, we subtract the background field energy from the total field energy and maximise the relative field energy as

2 $${\Delta}U_{{\mathrm {field}}} \approx \frac{{\varepsilon _0}}{2}{\int} {{\Delta}n^2({\mathbf{r}})|E_{{\mathrm {in}}}({\mathbf{r}})|} ^2{\mathrm {d}}{\mathbf{r}}$$

where Δ*n*^2^(**r**) = *n*^2^(**r**) – *n*_m_^2^, and *n*_m_ the medium RI. According to the Parseval theorem, the 3D summation of the input field intensity remains constant if the laser power is constant, that is, ${\int} {|E_{{\mathrm {in}}}({\mathbf{r}})|^2{\mathrm {d}}{\mathbf{r}}} = C$. With this, the Cauchy–Schwarz inequality dictates that Eq. (2) is maximised when |*E*_in_(**r**)|^2^ is proportional to Δ*n*^2^(**r**).

### Experimental setup

**Fig. 1:**
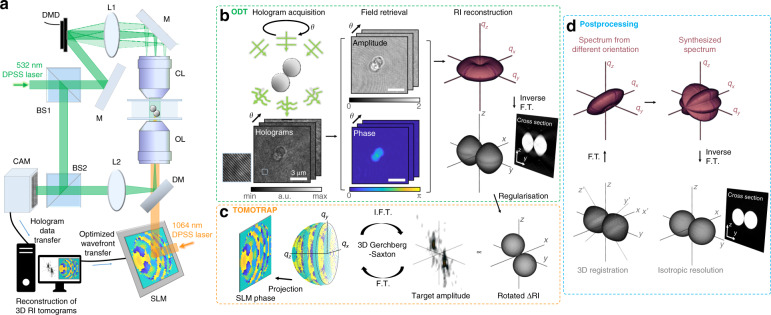
Experimental setup. **a** Optical setup (DMD digital micromirror device, L lens, M mirror, CL condenser lens, OL objective lens, DM dichroic mirror, BS beam splitter, CAM camera). **b** Optical diffraction tomography (ODT). The sample is illuminated at different incident angles, and the corresponding off-axis holograms are recorded. The raw holograms are converted to transmitted fields using the field-retrieval algorithm. The obtained fields are mapped to the Fourier space, and the inverse Fourier transform reconstructs the refractive index (RI) tomogram with low axial resolution. **c** Tomographic mould for optical trapping (TOMOTRAP). The phase distribution of the spatial light modulator (SLM) in the Fourier plane was optimised to make the light amplitude maximally overlap the regularised relative sample RI map via a 3D Gerchberg–Saxton (GS) iterative algorithm. **d** Postprocessing for isotropic 3D reconstruction. A reconstructed tomogram was registered with the initial raw RI tomogram in 3D to estimate the actual 3D orientation. Registration data were applied to obtain a spectrum from a different orientation. Finally, we obtain an isotropically synthesised tomogram and a spectrum devoid of the missing cone.

To experimentally demonstrate isotropic microtomography using TOMOTRAP, we combined optical diffraction tomography (ODT) for real-time RI microtomography^25^ and HOTs for simultaneous optical manipulation of a sample^16^ (Fig. 1 and the section “Materials and methods”). ODT is one of 3D quantitative phase imaging techniques, which reconstructs the 3D RI distribution of a sample from multiple 2D holograms measured at various illumination angles^2,25^ (Fig. 1b). Incident plane waves were scattered by the heterogeneous RI contrast of a sample and recorded as an interferogram. To extract transmitted complex fields from the raw holograms, we used a field retrieval algorithm based on the Fourier transform^26^. From the retrieved fields, we reconstructed the 3D RI map of the sample using the Fourier diffraction theorem with Rytov approximation^27,28^. Note the missing cone problem causes low axial resolution^3^.

HOTs implemented TOMOTRAP by experimentally generating the desired structured light in a real-time manner based on the measured RI tomogram (Fig. 1c). The target 3D amplitude of the light trap was the contrast of the reconstructed RI tomogram regularised by the nonnegativity constraint. We generated the light trap using a phase-only spatial light modulator (SLM) in the Fourier plane. We optimised the phase pattern of the SLM using a 3D Gerchberg–Saxton (GS) iterative algorithm.

After rotating the optically trapped sample to the desired orientation and recording its image, we reconstructed the isotropic RI tomogram in the postprocessing step (Fig. 1d). From the reconstructed RI tomogram of the optically rotated sample, we estimated the actual change in the orientation of the sample using an iterative 3D registration algorithm (see the “Materials and methods”). For a seamless isotropic reconstruction, we used the estimated registration data to obtain a rotated 3D sample spectrum. Followed by the repeated registration, we selected successful data to obtain a synthesised spectrum without the missing cone and a subsequently isotropic RI tomogram.

### Isotropic reconstruction of multimeric PMMA colloids

**Fig. 2:**
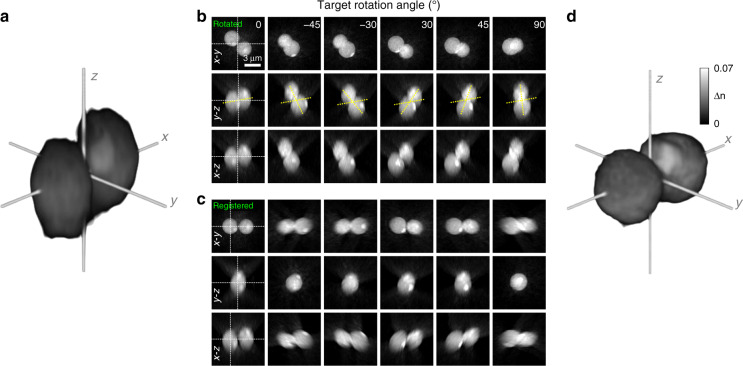
Validation of the isotropic reconstruction process using a standard sample. **a** Raw RI tomogram of a PMA dimer reconstructed by conventional ODT. **b**, **c** Sliced tomogram images of the sample rotated by TOMOTRAP. **b** Raw data. **c** Registered results. The lateral pitch axis of the raw data was rotated by 45° with respect to the *x*-axis, and registered to the *y*-axis. The yellow and white dashed lines indicate rotational angles and the cross-sections, respectively. **d** Isotropic RI tomogram obtained from the registered data.

We first validated the feasibility of the proposed method using a standard sample whose structure was simple, and the 3D RI distribution was well known. For this purpose, we tested the optical rotation of a colloidal suspension of 3 μm diameter poly(methyl acrylate) (PMA) bead dimers in a 70% aqueous solution of glycerol (Fig. 2a; see the section “Materials and methods” for sample preparations). To obtain a quasi-spherical spectrum with as small numbers of rotations as possible, we rotated the optically trapped particle to target angles varying from −45° to 90° with an interval of 15° (Fig. 2b). The 3D registration algorithm successfully estimated and corrected the actual 3D orientation of the optically rotated sample (Fig. 2c). The measured misorientation angles with respect to the lateral pitch axis of the sample were within ±4°, with the exception of 90° where the deviation exceeded 12°. We speculate that the highest angle deviation at the angle of 90° resulted as the particle was inclined towards the optical axis, where multiple light scattering is significant. Nevertheless, the misorientation was mitigated by the corrected registration data, and the resultant isotropic RI tomogram was successfully reconstructed (Fig. 2d). In visual inspection, the heterogeneous protrusions on the colloid were clearly reconstructed, which suggests that the reconstruction process can provide an isotropic RI tomogram with high fidelity.

**Fig. 3:**
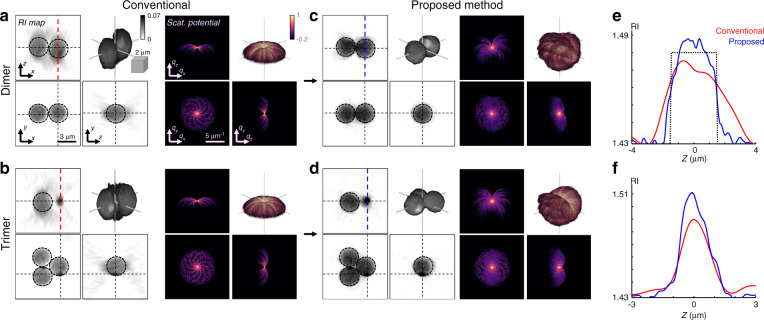
3D RI reconstruction of PMA multimers. **a, b** RI tomograms and the corresponding scattering potential spectra of a PMA (**a**) dimer and (**b**) trimer using the conventional ODT. **c**, **d** Corresponding results of the PMA (**c**) dimer and (**d**) trimer using our proposed method. (Dashed lines, 2D slice regions; inset, 3D-rendered images). **e**, **f** Line plots along the coloured dashed lines for (**e**) the dimer and (**f**) trimer (dashed circles, guiding lines for ideal 3 μm diameter spheres with RI of 1.48).

We examined the isotropic RI reconstruction performance for various colloidal suspensions (Fig. 3). We tested a PMA dimer and a trimer, and successfully rotated them by [−45°, −30°, 30°, 45°, 90°] and [−45°, −30°, 30°, 45°], respectively (see Fig. S1a, b). We compared the RI tomograms that were reconstructed from the conventional ODT without sample rotation with those from our proposed method, which exhibited marked differences. In the conventional ODT with the missing cone problem, both the PMA dimer and trimer exhibited axially elongated artefacts (Fig. 3a, b). In contrast, our proposed method using TOMOTRAP clearly reconstructed the spherical shapes of the 3 μm diameter multimers with improved RI contrasts (Fig. 3c, d). Interestingly, the sample spectra without the missing cone in our method revealed the interferometric signals that are characteristic of multimeric particles^29^. More importantly, the isotropic RI tomograms allowed enhanced recognition of the heterogeneous surfaces on the beads. These visual results highlight the importance of high-resolution assessment in microtomography.

To quantitatively analyse the enhanced axial resolution, we compared the axial RI profiles of the reconstructed colloidal particles (Fig. 3e, f). We first plotted the axial RI profiles along the centre of one of the monomers in the PMA dimer (Fig. 3e). In the conventional ODT, the RI profile was blurred along the axial direction and the RI value was underestimated compared with the nominal diameter of 3 μm and RI value of 1.476. On the other hand, the RI profile obtained from our proposed method closely agreed with the expected RI profile based on the nominal sample specifications, with a sharper full width half maximum (FWHM) of 3.3 μm and a higher peak RI value of 1.485. We next analysed the axial RI profiles of a heterogeneous protrusion observed in the PMA trimer (Fig. 3f). Consistent with the previous results, our proposed method improved the axial resolution and RI contrast of the singular structure. The estimated FWHM and peak RI value of the singular structure improved from 1.67 to 1.38 μm and from 1.49 to 1.51, respectively. Thus, we successfully verified the applicability of the proposed method for various multimeric colloidal suspensions.

### Isotropic reconstruction of the live mouse RBCs

Accurate 3D imaging and control of live RBCs are some of the most elusive tasks in microtomography. RBCs are susceptible to the missing cone problem owing to their axially thin biconcave structures. Moreover, they are easily deformed; therefore, rotating them without deformation requires careful sample manipulation. These shortcomings can be mitigated by employing TOMOTRAP.

**Fig. 4:**
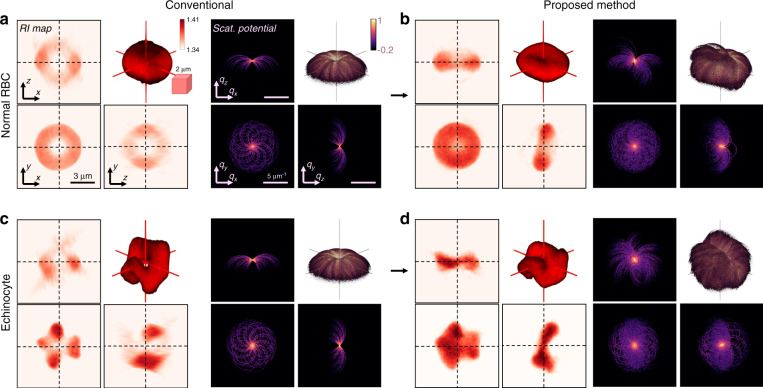
3D RI reconstruction of live mouse red blood cells (RBCs). **a**, **b** RI tomograms and the corresponding scattering potential spectra of (**a**) a normal RBC and (**b**) echinocyte using the conventional ODT. **c**, **d** Corresponding results of (**c**) the normal RBC and (**d**) the echinocyte using our proposed method (dashed lines, 2D slice regions; inset, 3D-rendered images).

We isotropically reconstructed the RI of two types of live mouse RBCs suspended in phosphate-buffered saline (PBS): a normal RBC and an echinocyte (Fig. 4; see the section “Materials and methods”). We successfully rotated the normal RBC by 45° and 60°, and the echinocyte by −60°, −45°, −30°, 30°, and 90° using TOMOTRAP, with minimal deformation and precise registration (see Fig. S1c). The effect of removing the missing cones was significant. The missing cone problem in the conventional ODT caused vacant artefacts at the centres of both cells, which are also observed in the simulations^30^ (Fig. 4a, b). However, our method resolved the biconcave dimples and folded structures of the cells (Fig. 4c, d). Notably, the estimated RI values of both samples reached a maximum of 1.39, without regularisation. The collective results suggest the general feasibility of TOMOTRAP for in situ isotropic microtomography of various freestanding specimens.

### Axial resolution analysis

**Fig. 5:**
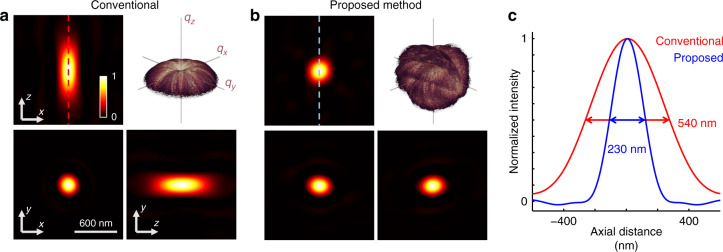
Resolution analysis. **a**, **b** Sliced images of the 3D CSFs obtained from (**a**) conventional ODT and (**b**) using our proposed method. Insets, coherent transfer functions used to define CSFs **c** Axial line profiles of the CSFs along the coloured lines in (**a**) and (**b)**. FWHM of the conventional ODT and our proposed method are indicated.

We conclude the analysis with a quantitative comparison of the axial resolution between the conventional ODT and the proposed method. We defined the axial resolution as the FWHM of a 3D coherent spread function (CSF), which is the inverse Fourier transform of the coherent transfer function (the bandwidth range of the 3D Fourier spectrum; Fig. 5). The analysis showed that the isotropic reconstruction of the PMA dimer exhibited the maximum improved axial resolution (Fig. 5a, b). Conventional ODT suffered from an axially elongated artefact in the obtained CSF; however, our proposed method remained unaffected. This was confirmed quantitatively by comparing the axial FWHMs of both methods (Fig. 5c). Our method provided 230 nm axial resolution, which was 2.36 times better than that of the conventional ODT (540 nm). In the experiments with the PMA trimer, normal RBCs, and echinocytes, the enhancement factors of the axial resolution were 1.99, 1.83, and 2.21, respectively (see Fig. S2). The overall results suggest that the axial image resolution can be significantly improved by implementing the proposed method.

## Discussion

In summary, we have used HOTs to demonstrate the stable rotation of freestanding microscopic specimens and in situ isotropic microtomography. By integrating ODT for RI tomography and TOMOTRAP for optimised optical trapping, we validated stable orientational control of samples varying from self-assembled colloidal suspensions which were used as the standard test samples, to live RBCs which were considered most challenging^30^. The corresponding isotropic tomograms captured the morphological details of these specimens in their native states with minimal deformation and damage. These results suggest that, as an alternative to the traditional methods of rotating fixed macroscopic samples, our proposed method can offer a general option for sample rotation tomography of free-floating microscopic samples.

The proposed method opens up the possibility of various applications in soft matter research and cell biology. The apposite applications are 3D inspection of floating microsystems with complex structures. In soft matter physics, the relevant examples are 3D imaging of aspheric colloids^31^, liquid crystal droplets, micro-antennas^32^, and crystalline particles^4,33^. In cell biology, the proposed method can be used for accurate 3D tracking and analysis of live blood cells^34^, bacteria^35^, and yeast cells.

The average execution time of the registration was 5 min per orientation, which makes the total required time for generating an isotropically resolved sample approximately an hour when 10 different tomograms were used. Future improvements would promote broader real-time applications of the proposed method with greater throughput and accuracy. The throughput depends mostly on the processing time, which can be readily optimised by automated processing and further accelerated by using a graphical processing unit (GPU) and introducing efficient algorithms for fast RI reconstruction^36^, registration, and pattern optimisation^37^. The accuracy relies on exact RI estimation and 3D registration in the presence of missing cones and multiple light scattering, which should be solved using advanced regularisation algorithms^3,38^ and inverse scattering problem solvers^39^, respectively. These computational approaches do not compete with our proposed method. Rather, they can be synergistically combined without changing the experimental setup to facilitate various future implementations.

We note that the range of applications is not limited to isotropic ODT. The proposed method is compatible with other ODT techniques that employ incoherent sources^40–42^. Besides RI tomography, it is also possible to retrieve the 3D absorptivity maps of chromophores^43^. Fluorescence^13^ and label-free nonlinear microscopy^44^ can also be integrated for correlative volumetric analyses of live cells^45^. Ultimately, the isotropic super-resolution tomography is expected to complement the conventional super-resolution fluorescence microscopy^46^.

## Materials and methods

### 3D GS algorithm

A light trap that satisfies the TOMOTRAP principle was generated by optimising the phase pattern of an SLM using a 3D GS algorithm. The phase-only SLM was placed in the Fourier plane and the wavefront of the trapping light was controlled. The bandwidth range of the light was confined within the Ewald cap, that is $A_{0}{({\rm{r}})} = \sqrt {n^{2}{({\rm{r}})} - n_{m}^{\,\,\,\,2}}$, where $(q_x^2 + q_y^2)$ ≤ (*q*_max_/*λ*_IR_)^2^, *q*_max_ = 1.3 (optical trap bandwidth NA), and *k*_IR_ = *n*_m, IR_/*λ*_IR_, where *n*_m, IR_ and *λ*_IR_ represent the medium RI and TOMOTRAP laser wavelength, respectively. The 3D target amplitude of the structured light in the image space was then set to the relative RI contrast, $A_{0}{({\rm{r}})} = \sqrt {n^{2}{({\rm{r}})} - n_{m}^{\,\,\,\,2}}$, where *n*(**r**) and *n*_m_ are the measured sample RI and the medium RI, respectively, at a wavelength of an ODT laser (see the section “Calibration of the medium RI” below). After initialisation, the GS algorithm starts with *p* = 0 (*p* represents iteration index). In the *p*th iteration, the phase of the SLM was set to $\tilde \phi _p({\mathbf{q}}) = {\mathrm {Arg}}[\widetilde A_p({\mathbf{q}})/q_z]$, where the amplitude *A*_*p*_(**q**) is divided by *q*_*z*_ to reflect the 2D projection geometry of the SLM. The 3D field generated by the SLM is given by: $B_p({\mathbf{r}}) = {\mathrm {IFT}}(q_z{\mathrm {exp}}[i\tilde \phi _p({\mathbf{q}})])$, where IFT is inverse Fourier transform. In the (*p* + 1)th iteration, the amplitude of the field was replaced with the target amplitude, $A_{p + 1}({\mathbf{r}}) = A_{\it{0}}({\mathbf{r}})\exp [i \cdot {\mathrm {arg}}\{ B_p({\mathbf{r}})\} ]$ and the iteration continued in steps of 1. To obtain a 3D sample mask, the parameters used for the optimal patterns were the iteration number of the GS algorithm, the iteration number of the non-negativity regularisation algorithm^47^, and the RI threshold. The iteration numbers were set to 40 and 25 for the GS algorithm and the non-negativity regularisation algorithm, respectively, throughout the study, and the RI threshold parameters were set to 1.44 and 1.36 for PMA multimers and live mouse RBCs, respectively. For a 256 × 256 × 256 voxelated tomogram, the average elapse time per iteration of the 3D GS algorithm without and with use of a GPU (NVIDIA GeForce GTX 1070 Ti) was 279.7 and 5.5 ms, respectively.

### Optical setup

The schematic is depicted in Fig. 1a. In the ODT, a plane wave from a continuous green laser (SambaTM 532 nm laser, maximum power = 100 mW, Cobolt) illuminated the sample. The green plane wave was obtained by spatial filtering with a collimating lens (*f* = 50 mm) and a 30-μm-diameter pinhole (P30D, Thorlabs) and magnification with an additional telescopic imaging system (*f* = 500 mm). After the plane wave was separated by a half-wave plate (WPH10M-532, Thorlabs) and the first polarising beam splitter (PBS251, Thorlabs), the incident plane at the sample path wave was diffracted by a digital micromirror device (DMD; DLP6500EVM, Texas Instruments) for high-speed angular scanning^48,49^, and angularly magnified by a 4-*f* array of lens 1 (*f* = 250 mm) and condenser lens (LUMFLN60XW, NA = 1.1, Olympus). An objective lens with a high NA (UPlanSApo100XO, NA = 1.4, Olympus) collected the transmitted sample field, and a tube lens 2 and an additional 4-*f* array magnified the image by 111.1 on a camera (MQ042MG-CM, Ximea). For the off-axis holography, a reference plane wave was combined with the sample field using the second polarising beam splitter and a linear polariser. For tomographic imaging, 61 holograms were recorded with different angles of incidence for the plane waves.

In the TOMOTRAP, a plane wave from a continuous high-power infra-red laser (1064-10-CW, wavelength = 1064 nm, maximum power = 10 W Coherent) was impinged on a liquid-crystal phase-only SLM (X10468-07, Hamamatsu) for wavefront modulation. The infra-red plane wave was obtained by spatial filtering with a collimating lens (*f* = 60 mm) and a 50-μm-diameter gold-plated pinhole (P50C, Thorlabs) and magnification with an additional telescopic imaging system (*f* = 500 mm). The linear polarisation of the plane wave was then modulated by an array of a linear polariser (LPNIRE100-B, Thorlabs) and a half-wave plate (WPH10M-1064, Thorlabs). The plane wave impinged onto the SLM, whose plane was conjugated to the pupil plane of the objective lens with a de-magnification ×1.3 of 4*-f* arrays. Among the 800 × 600 pixels of the SLM, a circular aperture with a radius of 153 pixels was used. The corresponding NA of the modulated beam was 1.3 in which the effect of aberration was negligible. The power of the trapping beam in the sample plane ranged from 1.35 to 4.05 mW, at which no significant light-induced sample damage was observed throughout the experiments. The paths of TOMOTRAP and ODT were combined using a dichroic mirror (DMSP950R, Thorlabs).

### Registration of the rotated tomograms

To accurately estimate the 3D orientation and displacement of an optically rotated sample, an RI map of the rotated sample was iteratively registered with the RI tomogram obtained before the optical trapping. Initially, the translational and rotational components of the rigid transformation matrix, *T*_0_, were estimated by 3D correlation analyses and target orientations, respectively. For a more accurate registration, we iteratively searched for an optimal set of error-correcting translation vectors and Euler rotation angles, (**t**, *ψ*, *θ*, *φ*), at which the rigid transformation $R({\mathbf{t}})R(\psi \hat z)R(\theta \hat x)R(\varphi \hat z)$ leads to the maximisation of the 3D Pearson correlation coefficient between the registered tomogram and the initial RI tomogram. During the registration sequence, the fitting range of the Euler rotation angles decreased from [−8°, 8°] to [−4°, 4°] and [−2°, 2°], and the fitting interval correspondingly decreased from 2° to 1° and 0.5°. This total registration sequence was iterated twice during the postprocessing step and took 281.9 s, which could be further accelerated by using a GPU.

### Calibration of the medium refractive index (RI)

We measured the medium RI values at blue (488 nm, 06-MLD, Cobolt), yellow (561 nm, MLL-FN-561, CNI Laser), and red (639 nm, MLL-FN-639, CNI Laser) laser wavelengths using a handheld refractometer (R-5000, Atago^TM^), and interpolated the dispersion relation using Cauchy’s equation. The estimated RI values of a 70% aqueous glycerol solution were 1.428 and 1.426 at 532 and 1064 nm wavelength, respectively. The estimated RI values of the PBS solution followed the reported RI values of water^50^.

### Sample preparations

The 3 μm diameter PMA bead multimers were prepared by drying a drop of the purchased suspension (86935-5ML-F, Sigma-Aldrich, USA) on a Petri dish for a day; followed by scratching and mixing the dried sample into a 70% aqueous solution of glycerol. The van der Waals attraction ensured that the aggregated beads would not disassemble after suspension. The colloidal suspension was then placed between two No. 1 thickness cover glasses (24 × 40 mm^2^, Matsunami, Japan), which were spaced apart by 100 μm-thick adhesive tapes. The blood extracted from a wild-type mouse through a syringe was immediately diluted in PBS to form an RBC solution, which was then poured on a Petri dish (Tomodish, Tomocube Inc.). To prevent the RBCs from adhering to the dish, the dish surface was coated with a 4% bovine serum albumin solution. The upper side of the dish was then covered with a No. 1.5H thickness coverslip (20 × 20 mm^2^, Marienfeld).

### Analysis

All computational reconstructions and analyses were performed using MATLAB 2019b. The 3D images were visualised by using the *volshow* function.

## Supplementary information

Supplementary Information
