# Supplementary material for: Isotropically resolved label-free tomographic imaging based on tomographic moulds for optical trapping
Source: Light Sci Appl. 2021 May 17;10:102. doi: 10.1038/s41377-021-00535-4 (PMC8126562; doi:10.1038/s41377-021-00535-4)
Supplement: Supplementary file 1 — Supplementary Information [file 41377_2021_535_MOESM1_ESM.docx]

**Supplementary Information for Isotropically resolved label-free tomographic imaging based on tomographic moulds for optical trapping**

Moosung Lee^1,2^, Kyoohyun Kim^1,3^, Jeonghun Oh^1,2^, and YongKeun Park^1,2,4*^

*1 Department of Physics, Korea Advanced Institute of Science and Technology (KAIST), Daejeon 34141, South Korea;*

*2 KAIST Institute for Health Science and Technology, KAIST, Daejeon 34141, South Korea;*

*3 Current affiliation: M**ax Planck Institute for the Science of Light & Max-Planck-Zentrum für Physik und Medizin, Erlangen 91058, Germany;*

*4 Tomocube Inc., Daejeon 34109, South Korea*

**corresponding authors: Y.K.P (*yk.park@kaist.ac.kr*)*

**E-mail addresses**

Moosung Lee: lkaamo@kaist.ac.kr

Kyoohyun Kim: kyoohyun.kim@mpl.mpg.de

Jeonghun Oh: jhun18@kaist.ac.kr

YongKeun Park: yk.park@kaist.ac.kr

**FIGURES**


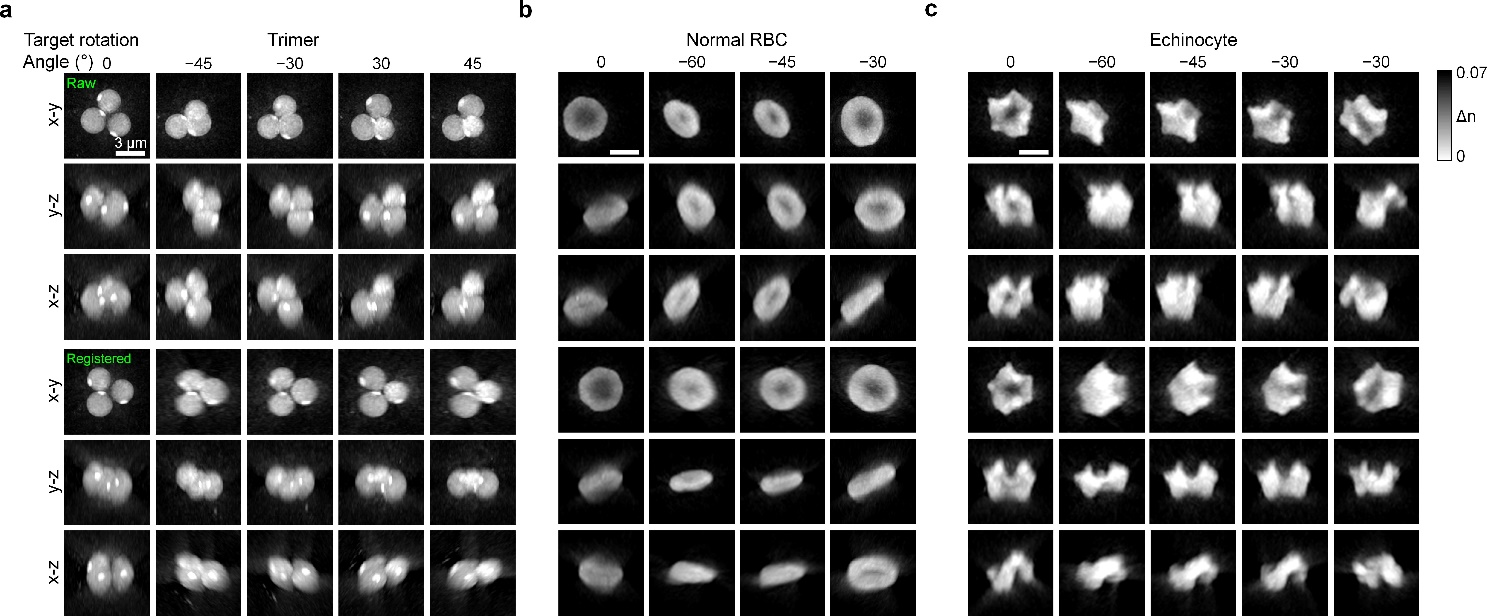


**Figure S1 | RI slice images of optically rotated samples and the registered results.**

**a,** 3-μm-diameter PMA trimer. **b, c,** Live mouse RBCs. **(b)** A normal RBC and **(c)** an echinocyte. The lateral pitch axis of each registered data was set to the y-axis.


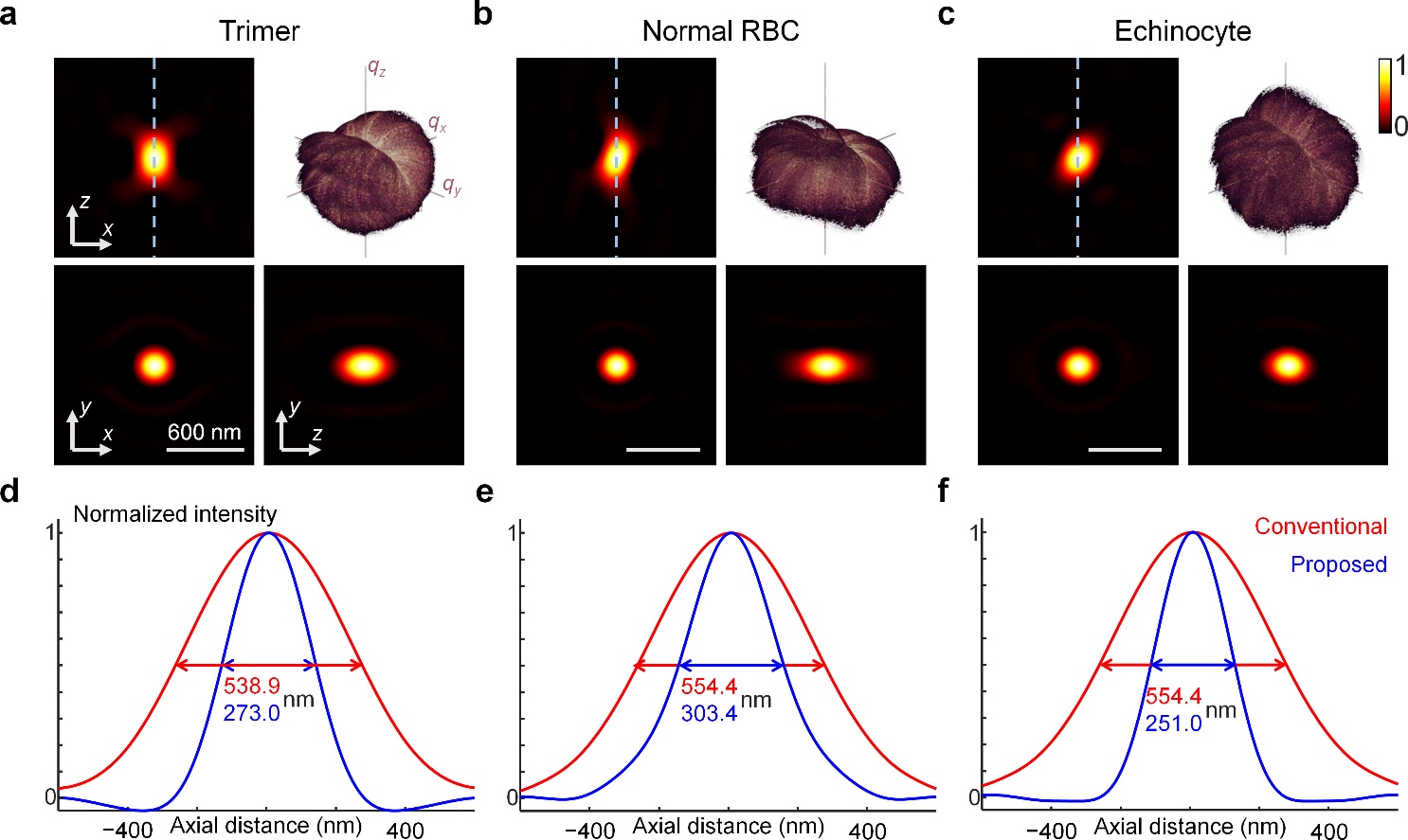


**Figure S2 | Resolution in different experiments**

**a-c,** Sliced images of 3D coherent spread functions (CSFs) for **(a)** a 3-μm-diameter trimer, **(b)** a normal mouse RBC, and **(c)** an echinocyte. **d-f,** Axial line profiles of CSFs along the coloured lines in **(a-c)**. Full-width half-maxima obtained from conventional ODT and our proposed method are indicated.
